# The relationship between smartphone addiction and sleep among medical students: A systematic review and meta-analysis

**DOI:** 10.1371/journal.pone.0290724

**Published:** 2023-09-15

**Authors:** Mabel Qi He Leow, Joelle Chiang, Tiffany Jia Xuan Chua, Sean Wang, Ngiap Chuan Tan

**Affiliations:** 1 Singhealth Polyclinics, Singapore, Singapore; 2 Lee Kong Chian School of Medicine, National Technological University of Singapore, Singapore, Singapore; 3 Yong Loo Lin School of Medicine, National University of Singapore, Singapore, Singapore; 4 SingHealth Duke-NUS Family Medicine Academic Clinical Program, Duke-NUS Medical School, Singapore, Singapore; McMaster University, CANADA

## Abstract

**Objectives:**

This systematic review aimed to evaluate the association between smartphone addiction and sleep in medical students. The secondary outcomes included the prevalence of smartphone addiction, duration and purpose of its use, prevalence of poor sleep, duration and quality of sleep.

**Methods:**

The authors searched PubMed, Cochrane Library, Embase, PsycINFO and CINAHL databases, from inception of each database to October 2022. Quantitative studies in the English language on smartphone addiction and sleep in students studying Western Medicine were included. The Rayyan application was used for title-abstract screening, and Joanna Briggs Institute (JBI) critical appraisal checklist to assess the risk of bias. Heterogeneity tests and meta-synthesis of data were performed using the meta-package in R software. Data on the activities used on the smartphone was synthesized qualitatively

**Results:**

A total of 298 abstracts were initially assessed for inclusion eligibility: 16 of them were eventually appraised, covering 9466 medical students comprising 3781 (39.9%) males and 5161 (54.5%) females. Meta-correlation between the Smartphone Addiction Scale Short Version (SAS-SV) and Pittsburgh Sleep Quality Index (PSQI) was 0.30 (95%CI = 0.24–0.36), and 0.27 (95% CI = 0.18–0.36) for SAS-SV and sleep duration. The meta-analytic estimation of smartphone addiction prevalence was 39% (95%CI = 0.30–0.50), and score using SAS-SV was 31.11 (95%CI = 29.50–32.72). The mean duration of smartphone daily used was 4.90 hours (95%CI = 3.72–6.08). The meta-analytic estimation on prevalence of poor sleep was 57% (95%CI = 0.48–0.66), and the meta-mean of PSQI and duration of sleep was 5.95 (95%CI = 4.90–7.00) and 5.62h (95%CI = 4.87–6.36) respectively. Medical students used their smartphones mostly for text messaging, followed by photo-sharing or social networking. Its usage for medical education remains unclear.

**Conclusion:**

The prevalence of poor sleep and smartphone addiction in medical students was 57% and 39% respectively, with a correlation index of 0.30. Medical students commonly used the smartphone for text-messaging, photo-sharing or social networking, averaging 4.9 hours daily.

## Introduction

Smartphones have become an inextricable part of daily life with the advancement of mobile technology and digitization, with 6.64 billion (83.32%) of the global population owning at least one device in 2022 [1]. In Singapore, 5.17 million people use smartphones, with its usage achieving 100% in youths aged 15 to 24 years old [2]. From 2019 to 2021, its local population aged 16–64 years spent an average of 3.6 hours on the smartphone internet daily [3].

Smartphone addiction was defined as users underestimating the amount of time spent on their smartphones, or being unable to regulate their use, resulting in negative consequences in daily life [4, 5]. Overuse of smartphones can result in addiction [6]. A multi-national study by conducted by Pew research center to understand the activities performed on a smartphone [7], and found that social activities were most common (i.e. sending text messages taking pictures or videos, and posting on social media), followed by seeking health and education related information.

Smartphone addiction can result in poor sleep quality, reduced sleep duration, and daytime dysfunction [8–11]. An association between smartphone addiction and poor sleep quality in university students and young adults has been observed [12, 13]. In a cross-sectional study which involved 1043 young adults aged 18–30 years in London, 68.7% of them had both smartphone addiction and poor sleep, reflecting a prevalent problem [13].

Poor sleep can impact both mental and physical health. Poor sleep quality increases risk for anxiety, suicide and depression [14–16]. In the longer-term, poor or inadequate sleep may result in musculoskeletal disorders such as cervical disc degeneration or hand joint inflammation [17], or chronic diseases such as hypertension, obesity, type-2 diabetes mellitus, cardiovascular disease, neurodegeneration, and dementia [18]. Compared with students from other degree courses such as law and economics, who also faced high academic demands, medical students had poorer sleep [19]. Poor sleep quality in medical students can also affect academic performance [20].

Smartphone addiction can have profound impacts on medical students’ mental health [21] and academic performance [22–24]. Zhong *et al*. [21] conducted a systematic review on the impact of smartphone addiction among Asian medical students on their mental health. From the nine studies from 2014 to 2019, 41.9% of Asian medical students had smartphone addiction. Smartphone addiction also positively correlated with poor sleep quality, stress, anxiety, depression, neuroticism, and poor general health. In medical students, inadequate sleep can cause emotional exhaustion, and the latter has a statistically significant relationship with decreased academic performance [22]. Smartphone addiction resulted in poorer academic performance in two studies on university students [23, 24]. Conversely, Boonluksiri [25] reported that even though smartphone addiction was associated with increased napping during class among medical students, their academic performance grading was unaffected.

Both poor sleep and smartphone addiction can result in poor health and academic performance. Thus, this systematic review aimed to evaluate the association between smartphone addiction and sleep in medical students. The secondary outcomes included the prevalence of smartphone addiction, duration of smartphone use, prevalence of poor sleep, duration of sleep, quality of sleep using a sleep survey, and type of activities medical students perform on the smartphone.

## Methods

### Literature selection

The systematic review was carried out according to the PRISMA statement and reported with the PRISMA checklist [26]. A priori protocol is registered on PROSPERO (CRD42022370847). Quantitative studies which used an observational design (cohort, case-control) or descriptive studies design were included. The review excluded publications covering reports, expert opinion papers, narratives, commentaries. Eligibility criteria included English studies which focused on smartphone addiction and sleep in students training in undergraduate or postgraduate Western Medicine.

### Information sources & search strategy

A three-step search strategy was utilized in this review. An initial limited search of MEDLINE and CINAHL was undertaken followed by analysis of the text words contained in the title and abstract, and of the index terms used to describe the articles. A second extensive search using all identified keywords and index terms was then undertaken across all included databases to further identify articles. During the conduct of the search, consideration was given to the diverse terminology used and the spelling of keywords as it might influence the identification of relevant studies. Finally, a hand search of the reference lists and bibliographies of retrieved articles was conducted to search for additional published studies not located through the databases that were used. Only published English papers were included.

A literature search of the databases was conducted from the date of inception of each database to October 2022. The electronic databases included were PubMed, Cochrane Library, Embase, PsycINFO and CINAHL (Cumulative Index to Nursing and Allied Health Literature). A search strategy based on the MeSH headings for these terms were used: ‘medical students’, ‘smartphone usage’, ‘screentime’, ‘smartphone addiction scale’, ‘problematic use of mobile phones’, ‘mobile phone dependence’, ‘app usage tracker’, ‘android application quality time’, ‘sleep quality’, and ‘sleep duration’. A combination of these terms were used for all the databases. The search strategy is in Appendix 1 in S1 File.

### Quality assessment and characteristics of included studies

Two reviewers were involved throughout the process of title-abstract screening, full-text screening and appraisal of the selected studies. Blinding between the two reviewers was ensured throughout the process (ML and JC) by conducting the assessments separately. Any disagreements between the two reviewers was resolved through discussion with a third reviewer (NCT).

The Rayyan application was used for title-abstract screening, followed by extraction of the full-text papers for review. Papers which meet the review’s inclusion criteria were appraised. The Joanna Briggs Institute (JBI) critical appraisal checklist (observational design or descriptive design tool for the respective types of studies) was used to assess the risk of bias. Decisions were recorded on Microsoft excel spreadsheet.

All the relevant data from the studies included were entered into an excel spreadsheet (authors, year of publication, country, study design, study setting, participants’ characteristics, number of participants, participants’ demographics, outcome measures, and results of study). For studies with missing data, the study team contacted the authors for unreported data or additional details.

Sleep could be measured by sleep duration, or a sleep tool such as the Pittsburgh Sleep Quality Index (PSQI), Athens Insomnia Scale (AIS), Insomnia Severity Index (ISI), or the PROMIS Sleep Disturbance scale. Smartphone addiction could be measured scales such as the Smartphone Addiction Scale (SAS), Cell-Phone Overuse Scale (COS), Problematic Mobile Phone Use Questionnaire (PMPU-Q) and Android Application Quality Time.

### Synthesis methods

The meta package in the R software was used for meta-synthesis of data [27, 28]. The main objective was to evaluate the association between smartphone addiction and sleep in medical students. Correlation coefficient was used for pooling of data. For studies which reported the odds ratio, this value was converted into a correlation coefficient to allow pooling of data [29].

Pooling of data was also performed for the prevalence of smartphone addiction and poor sleep, mean values obtained from the duration of sleep and smartphone use, and the scores from the sleep tool and smartphone addiction tool. If a study provided a median score, this was converted to mean and standard deviation based on a formula by Hozo and colleagues in 2005 [30]. Both the mean and standardised mean difference (SMD) were reported. The random-effect model was used if the heterogeneity (I^2^ statistic) was higher than 50%; otherwise, the fixed-effect model would be used [31]. Funnel plot asymmetry tests was conducted for outcomes which included more than 10 studies to ensure sufficient power for distinguishing chance from real asymmetry [32, 33].

The types of activities and smartphone applications used by the medical students were presented in percentages.

## Results

### Identified studies and quality assessment

**Fig 1:**
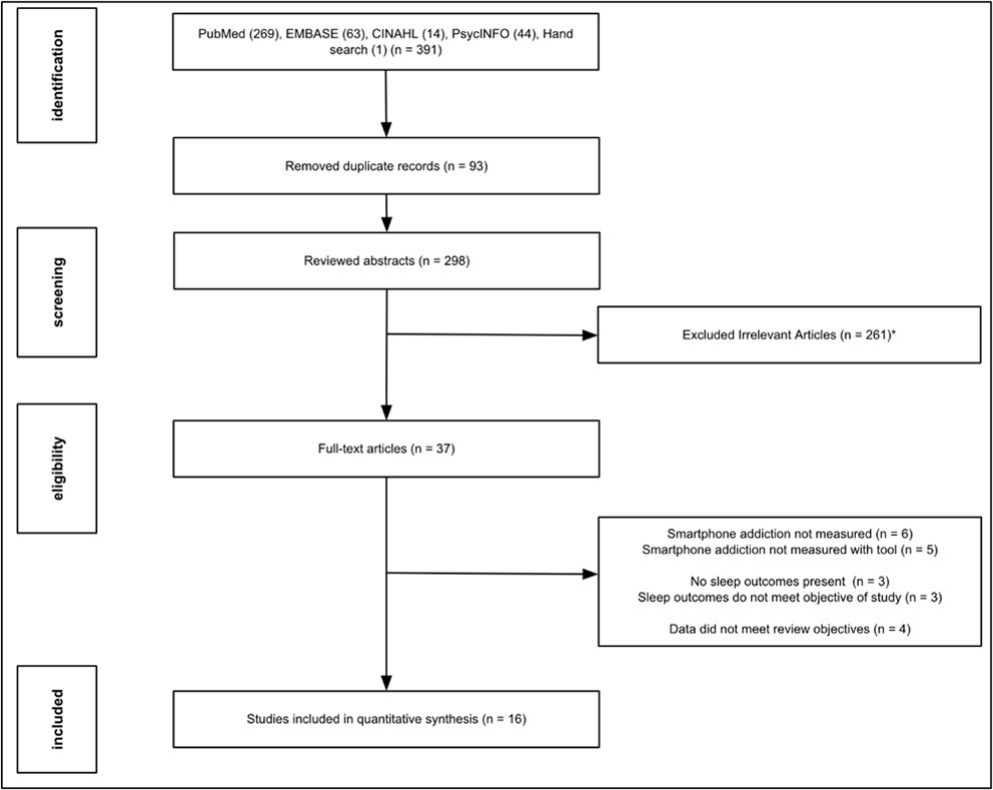
Flow chart and results of literature review for systematic review on the association between smartphone usage and sleep among medical students.

Three hundred and ninety papers were initially identified through a search of the literature from the database, of which 93 were duplicates. One additional paper was found using hand search. 298 abstracts were reviewed, and 261 papers were removed after evaluation of the abstracts. 37 studies were retrieved for detailed examination. 24 papers were excluded for the following reasons: smartphone addiction was not measured in six papers, smartphone addiction was not measured with a tool in five papers, no sleep outcomes were measured in three papers, sleep outcomes measured did not meet objectives of the review in three papers, and four papers did not provide data to meet the review’s objectives. Sixteen papers were critically appraised using the Joanna Briggs Institute (JBI) critical appraisal checklist for descriptive studies, and all papers were included in this review (Fig 1). The study data was reposited in Figshare [34].

**Fig 2:**
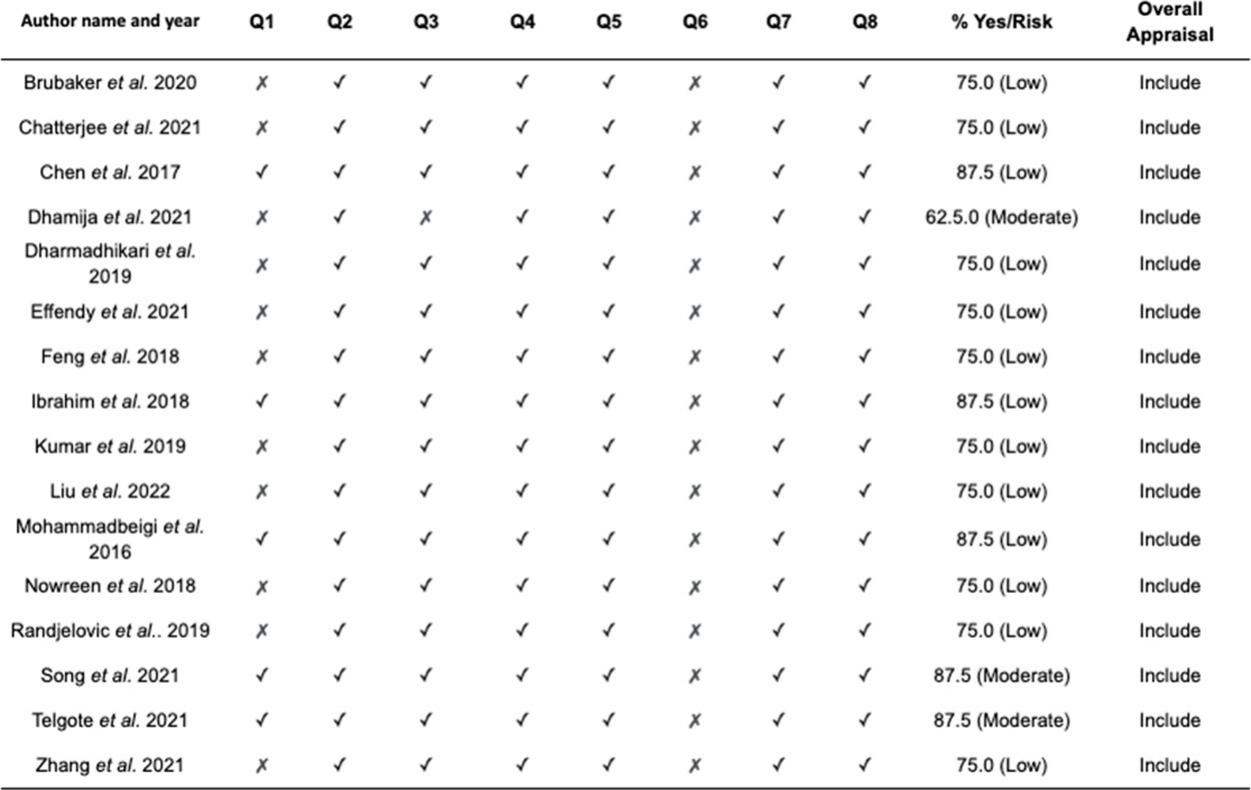
Appraisal of papers using Joanna Briggs Institute (JBI) checklist.

Fig 2 presents the appraisal using JBI checklist. Of the 16 papers reviewed, 13 had low risk of bias, and three had moderate risk for bias. Five studies used a random sampling method, while the other 11 studies used convenience sampling. The inclusion criteria were clearly defined for all the studies. Confounding factors were identified in 15 papers. All studies assessed outcomes using objective criteria, and if comparisons were made, there was sufficient description of the groups. All studies used a cross-section design, with no follow-up. Outcomes were measured in a reliable way for all papers, with appropriate statistical analyses being used.

### Profiles of the study population

The studies were published between 2016 and 2022, and conducted in India (n = 6), China (n = 5), Indonesia (n = 1), Iran (n = 1), United States of America (n = 1), Saudi Arabia (n = 1) and Serbia (n = 1). Among the combined population of 9466 medical students, 3781 (39.9%) were males and 5161 (54.5%) were females. Fourteen studies surveyed undergraduate medical students, and graduate medical students were the study populations of the remaining two studies. The profile of the study population is in Table 1.

**Table 1:** Profiles of the study population.

Author	Country	Participant (N)	Males (n)	Females (n)	Age in mean (SD)	Study population of Medical students	Sleep tool	Smartphone addiction tool
Nasution 2021 [38]	Indonesia	200	83	117	Median 21 Range (18–24)	Undergraduates	NIL	Indonesian SAS-SV
Nowreen 2018 [8]	India	212	106	106	19.8	Undergraduates	PSQI	SAS-SV
Mohammadbeigi 2016 [35]	Iran	280	116	164	21.8 (3.2)	Undergraduates	PSQI	COS
Kumar 2019 [10]	India	150	62	88	NR	Undergraduates	PSQI	SAS-SV
Brubaker 2020 [38]	USA	385	175	210	25.0 (2.4)	Graduates	PSQI	SAS-SV
Dharmadhikari 2019 [39]	India	195	96	99	20.2 (1.6)	Undergraduates	PSQI	SAS-SV
Zhang 2021 [61]	China	1016	354	662	26.0 (2.5)	Graduates	AIS	SAS-SV
Dhamija 2021 [62]	India	500	NIL	NIL	20	Undergraduates	PSQI	SAS-SV
Song 2021 [63]	China	666	262	404	47.3% age< 20, 52.7% >/ = 20	Undergraduates	PROMIS Sleep Disturbance Scale	SAS-SV
Ibrahim 2018 [36]	Saudi Arabia	610	314	296	21.6 (2.0)	Undergraduates	PSQI	PMPU-Q
Chatterjee 2021 [11]	India	224	104	120	21.1(1.79)	Undergraduates	PSQI	SAS-SV
Randjelovic 2019 [9]	Serbia	21	NIL	NIL	20–22 (range)	Undergraduates	PSQI	Android Application Quality Time
Chen 2017 [64]	China	1441	696	745	19.72 (1.4)	Undergraduates	PSQI	SAS-SV
Feng 2022 [43]	China	593	174	276	NR	Undergraduates	PSQI	MPAI Chinese version
Liu 2022 [65]	China	2741	955	1786	NR	Undergraduates	AIS	SAS
Telgote 2021 [66]	India	325	143	132	20.52(1.34)	Undergraduates	ISI	SAS-SV

Note. SAS-SV = Smartphone Addiction Scale–Short Version; PSQI = Pittsburgh Sleep Quality Index; COS = Cellphone Overuse Scale; AIS = Athens Insomnia Scale; PROMIS = Patient- Reported Outcomes Measurement Information System; PMPU-Q = Problematic Mobile Phone Use Questionnaire; MPAI = Mobile Phone Addiction Index; ISI = Insomnia Severity Index

#### Sleep

**Fig 3:**
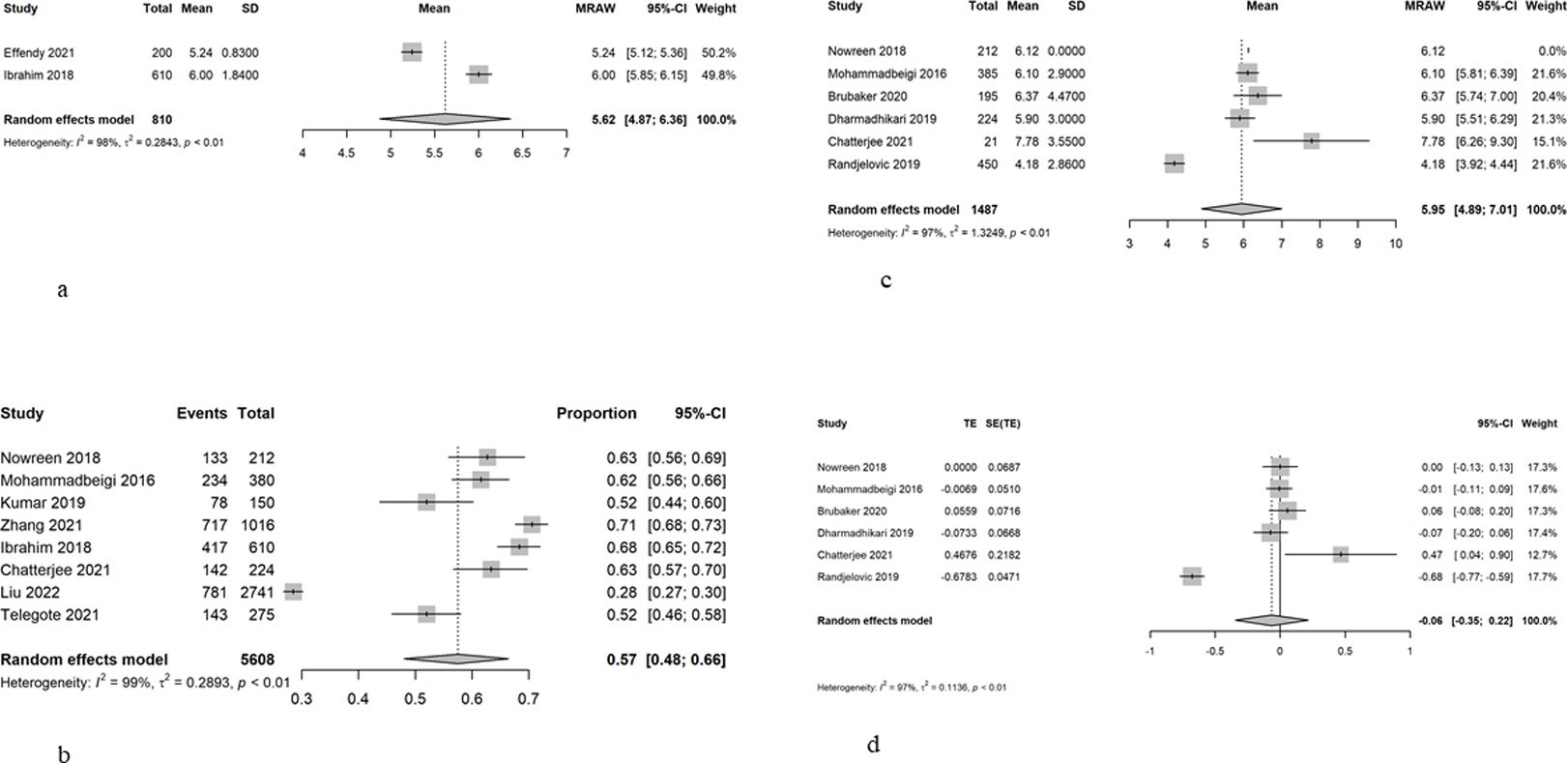
a. Mean sleep duration. b. Prevalence of poor sleep. c. Mean sleep score using the Pittsburgh Sleep Quality Index (PSQI). d. Standardised mean difference of sleep score using the Pittsburgh Sleep Quality Index (PSQI).

A sleep tool was used to measure sleep in 15 studies, including the PSQI (n = 11), AIS (n = 2), ISI (n = 1) and PROMIS (n = 1). One study measured sleep duration. The mean sleep duration from two studies is 5.62 (95%CI = 4.87–6.36), with a high heterogeneity of 98% (Fig 3A). The prevalence of poor sleep was 0.57 (95%CI = 0.48–0.66), with high heterogeneity of 97% (Fig 3B). A meta-mean of sleep score using the PSQI by six studies was 5.95 (95%CI = 4.90–7.00), with a high heterogeneity of 97%. When the non-Asian countries were excluded [35, 36], the heterogeneity was still high (97.6%), and random effects of 0.55 (95% CI = 0.43–0.66) (Fig 3C). Results from the SMD also showed high heterogeneity (97%), and random effects was -0.06 (95% CI = -0.35–0.22) (Fig 3D).

### Smartphone addiction

**Fig 4:**
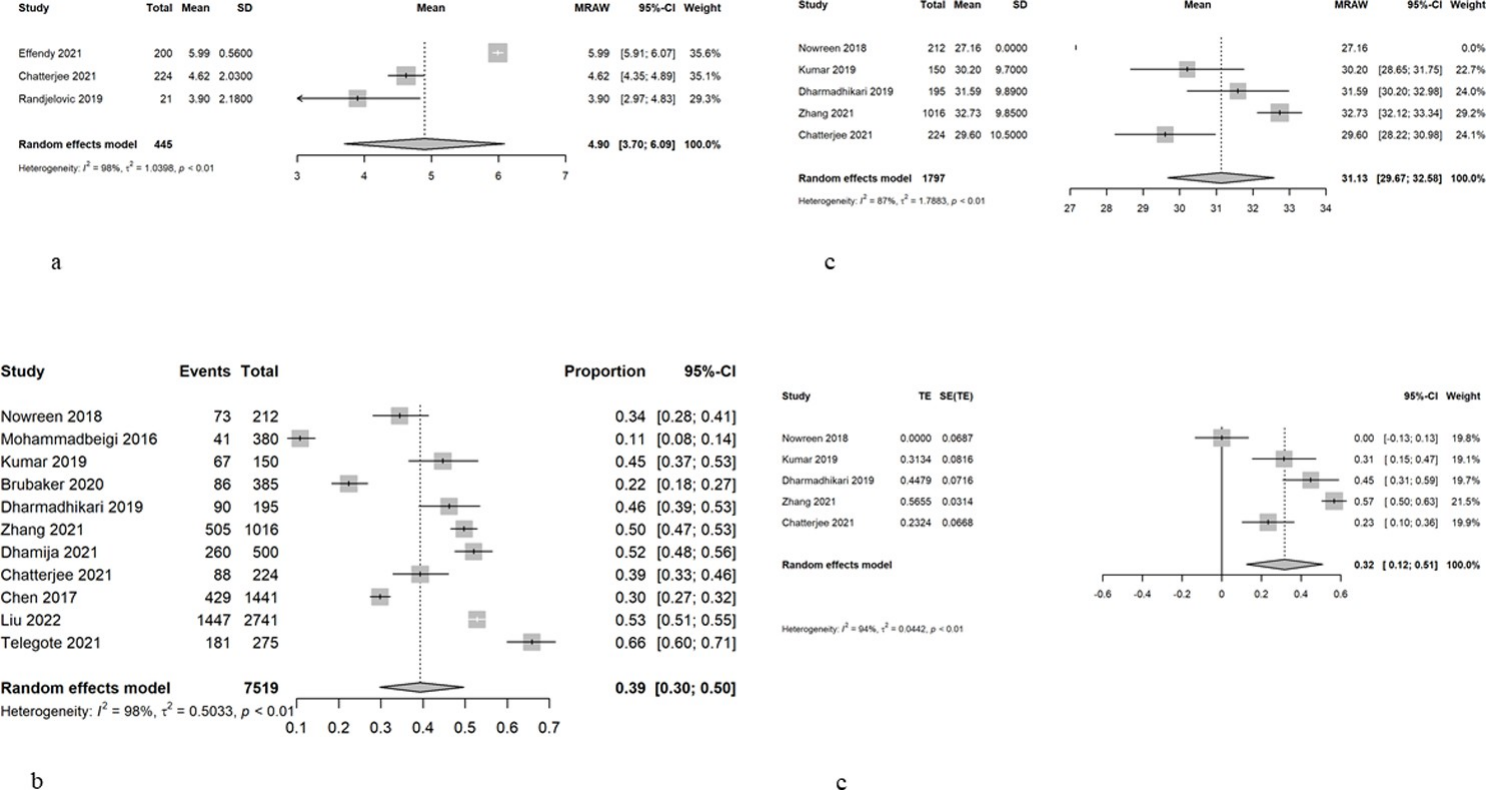
a. Duration of smartphone use daily.b Prevalence of smartphone addiction/overuse. c Smartphone addiction score using the Smartphone Addiction Scale Short Version (SAS-SV). d. Standardised mean difference of smartphone addiction score using the Smartphone Addiction Scale Short Version (SAS-SV).

Smartphone addiction was most commonly measured using the SAS-SV (n = 12). Other scales used included the COS (n = 1), PMPU-Q (n = 1), AAQT (n = 1), and MPAI (n = 1). The duration of smartphone daily used was described in 3 studies, with a meta mean of 4.90 (95%CI = 3.72–6.08) and high heterogeneity of 98% (Fig 4A). The overall prevalence of smartphone addiction in this study was 0.39 (95%CI = 0.30–0.50), with high heterogeneity of 99% (Fig 4B). Funnel plot test showed no publication bias (t = -1.25, p = 0.24). Excluding the non-Asian countries [35, 37], the prevalence was higher at 0.46% but the heterogeneity remained high at 96%. A meta-mean of smartphone addiction score using SAS-SV by five studies was 31.11 (95%CI = 29.50–32.72), with high heterogeneity of 87% (Fig 4C). Results from the SMD also showed high heterogeneity (94%), and random effects was 0.32 (95% CI = -0.12–0.51) (Fig 4D).

### Relationship between smartphone addiction and sleep duration

**Fig 5:**
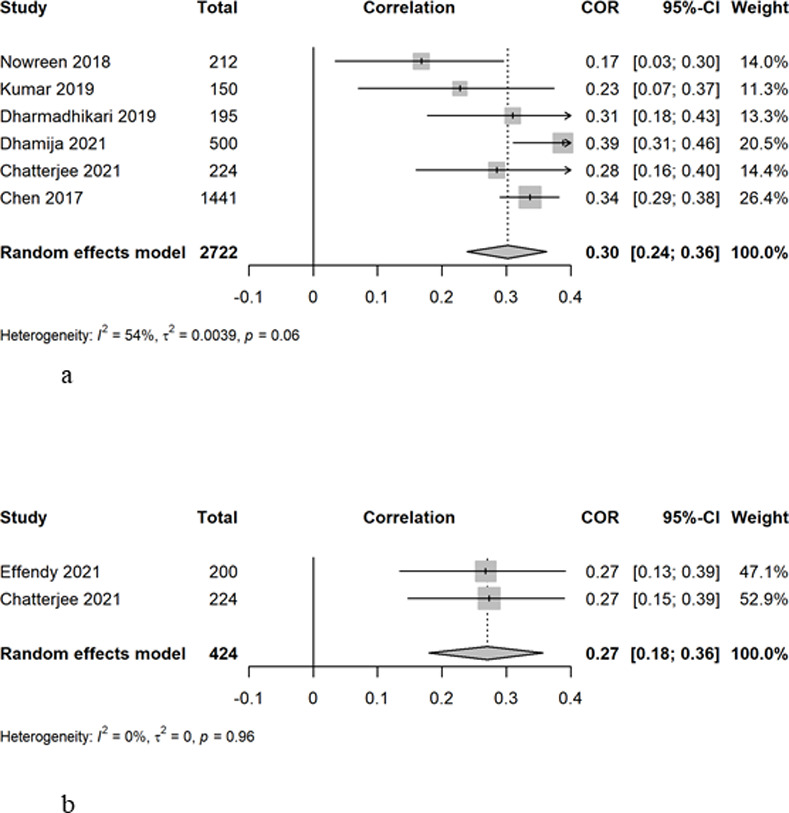
a. Correlation between Smartphone Addiction Scale Short Version (SAS-SV) and sleep duration. b. Correlation between Smartphone Addiction Scale Short Version (SAS-SV) and Pittsburgh Sleep Quality Index (PSQI).

Chatterjee^11^ reported a significant relationship between reduced sleep duration and increased duration of smartphone use (r = 0.18, p<0.001). Two studies analyzed the association between Smartphone addiction using SAS-SV and sleep duration. The overall correlation was 0.27 (95% CI = 0.18–0.36), with a low heterogeneity between studies of 0% (Fig 5A).

All studies except those by Nasution [38] and Ibrahim [36] tested the association between smartphone addiction and sleep scores. Six studies used both SAS-SV tool to measure smartphone addiction and PSQI to measure sleep, with either odds ratio or correlation coefficient values provided. The random effect of the correlation was 0.30 (95%CI = 0.24–0.36), with moderate heterogeneity of 54% (Fig 5B).

### Activities that students perform on smartphone

Three studies described the type of smartphone applications used (Table 2) [35, 36, 39]. The applications included Whatsapp, Instagram, Line, Snapchat, Viper, Tango, Path, Facebook, and YouTube. Whatsapp was the most common application used in all three studies. Instagram and Facebook were used in two studies. Text messaging was the most common activity performed on the smartphone in all three studies. This was followed by photo sharing or social networking.

**Table 2:** Activities performed on smartphone.

	Mohammadbeigi 2016 [35]	Dharmadhikari 2019 [39]	Ibrahim 2018 [36]
**Text messaging**			
Whatsapp (%)	60.9	33.9	45.0
Line (%)	35.5		
Viber (%)	58.1		
**Photo sharing**			
Instagram (%)		5.1	19.0
Snapchat (%)			35.0
**Social networking**			
Tango (%)	20.1		
Path (%)			11.0
Facebook (%)		12.3	7.0
**Videos**			
Youtube (%)			25.0

## Discussion

This systematic review investigated the association between sleep and smartphone addiction in medical students. The studies reported a significant correlation between sleep scores and smartphone addiction scores, sleep duration and smartphone addiction scores, and duration of sleep and smartphone usage. The overall correlation between SAS-SV and PSQI in this study was considerate low (r = 0.3). A previous study reported that the general population with problematic smartphone usage had a 2.19 odds ratio (estimated correlation coefficient = 0.211) of having poor sleep quality compared to those without [16]. Compared to the general population, this study found a higher correlation between smartphone addiction and sleep in medical students.

The prevalence of poor sleep scores in this review was 57%, which meant that over half of the medical students had poor sleep. This finding was almost similar to a previous study which described that 55% of medical students had poor sleep [40]. The mean PSQI in this review was 5.95, which was similar compared with to a systematic review study which reported a PSQI score of 6.1 in medical students [41]. Poor sleep seems pervasive amongst medical students. It can be attributed to stress, high study load, longer study times, studying prior to sleep, irregular work/rest schedules and anxiety about their studies and results [42–44]. Medical students were more anxious about their studies and spent less time on leisure activities compared to students in law and economics [42]. Poor sleep can also be attributed to low levels of sleep knowledge [45–47]. Earlier bedtimes and higher sleep duration during weekdays was associated with excellent academic performance [48]. However, Cvejic *et al*. [49] alluded that consistently short but unrefreshing sleep was associated with achieving higher academic grades.

The prevalence of smartphone addiction in this systematic review was 39%. This was lower than a previous study solely on Asian medical students which reported a prevalence of 41.9% [21]. When non-Asian countries (USA and Iran) were excluded, the prevalence was 46%, suggesting higher prevalence of smartphone addiction in Asian medical students compared to medical students from other regions. Higher prevalence of smartphone addiction in medical students was noted when compared to those studying other healthcare related subject. For example, the prevalence of smartphone addiction in nursing students was lower at 22% [50].

Qualitative synthesis revealed that text messaging was the most common activity performed on the smartphone, followed by photo sharing or social networking. Whatsapp was the most common text messaging platform. However, results from this review did not reflect the use of smartphone for academic purposes. A study conducted at an Indian medical college reported that 90% of the medical students across all years of studying identified smartphones to be useful for medical education, communication and instant access to information during bedside teaching [51]. A significant proportion of medical students used smartphone applications for online textbooks (70%), medical podcasts (60%), medical calculator (75%), online lecture (50%) and notes taking (45%) [52, 53]. However, smartphone applications such as Whatsapp had no impact on some medical students’ academic performance, but resulted in smartphone addiction and reduced sleep duration [54]. These applications could also cause superficial learning and distraction from textbooks [55].

### Strength and weakness of included studies

This systematic review aimed to include global studies. However, the majority of the studies were conducted in India and China, which are Asian countries. There was a lack of representation in studies conducted in the Western region. The studies covered largely undergraduate medical students, with a lack of studies which included graduate medical students to allow for comparison between both groups. The studies were largely descriptive cross-sectional surveys, with a high risk of bias. High heterogeneity was found in the results for both sleep and smartphone addiction, which could limit the reliability of the studies. The PSQI and SAS-SV were most commonly used in the studies. The remaining studies which deployed different sleep scales and smartphone addiction scales limited the pooling of data. None of the studies revealed the length of time which students used their phone to read medical materials or attend lessons remotely.

### Implications for policy, practice and research

Smartphone addiction correlated with poor sleep, with a high prevalence for both outcomes. It is important to create awareness on how overuse of smartphone can lead to smartphone addiction and reduced sleep, and to encourage medical students engage in physical activities instead of spending all their free time on the smartphone. Inculcating habits such as setting the phone to silent, do not disturb, or airplane mode during bedtime can help to prevent use and sleep disruptions. A systematic review recommended that exercise interventions, psychological interventions, cognitive behavioral therapy, and education program could be used to address smartphone addiction [56, 57]. For psychological interventions, Lan and colleagues [58] proposed that mindfulness helped to alleviate smartphone addiction by reducing desire for smartphone use and relieve their discomfort when required to leave their smartphone through cognitive reconstruction. Cognitive behavioral therapy may also be able to lower the desire to use a smartphone among people with smartphone addiction by reducing depression, impulsivity, anxiety [59, 60].

Future research on the use of smartphone in medical students can endeavor to uncover the number of hours the students spent studying on their phones. Longitudinal cohort studies can be conducted to determine if there is relationship between students’ academic performance with smartphone addiction and sleep.

## Conclusion

The prevalence of poor sleep and smartphone addiction in Medical students was 57% and 39% respectively, with a low correlation of 0.3. The prevalence of smartphone addiction in Asian medical students was 46%. They commonly use their smartphones for text messaging, photo sharing or social networking, with Whatsapp being the most common text messaging platform.

## Supporting information

S1 Checklist PRISMA 2020 checklist. (PDF)

S1 File Search strategy. (DOC)
