# Supplementary material for: The relationship between smartphone addiction and sleep among medical students: A systematic review and meta-analysis
Source: PLoS One. 2023 Sep 15;18(9):e0290724. doi: 10.1371/journal.pone.0290724 (PMC10503710; doi:10.1371/journal.pone.0290724)
Supplement: S1 File — (DOC) [file pone.0290724.s002.doc]

| **Search** **strategy** | |
| --- | --- |
| **PubMed** | ((medical students[Mesh terms]) OR (medical students[Title/Abstract]) OR (medical undergraduates[Mesh terms]) OR (medical undergraduates[Title/Abstract]) OR (medical graduates[Title/Abstract]) OR (student[Title/Abstract]))  AND  ((smartphone usage[Title/abstract]) OR (screentime[Title/abstract]) OR (Smartphone Addiction Scale[Title/abstract]) OR (Problematic Use of Mobile Phones[Title/abstract]) OR (Mobile Phone Dependence[Title/abstract]) OR (App Usage Tracker[Title/abstract]) OR (Android Application Quality Time[Title/abstract]) OR (Daily-life disturbance[Title/abstract]) OR (Withdrawal[Title/abstract]) OR (Cyberspace-oriented relationship[Title/abstract]) OR (Overuse[Title/abstract]) OR (Tolerance[Title/abstract]) OR (Prohibited use[Title/abstract]) OR (Dangerous use[Title/abstract]) OR (Dependent use[Title/abstract]) OR (Financial problems resulting from use[Title/abstract]) OR (Smartphone usage duration[Title/abstract]) OR (Tolerance and interference with other activities[Title/abstract]) OR (Abstinence syndrome[Title/abstract]) OR (Impaired impulse control[Title/abstract]) OR (Problems derived from use[Title/abstract]))  AND  ((sleep quality[Mesh terms]) OR (sleep quality[Title/abstract]) OR (sleep duration[Title/abstract]) OR (Pittsburgh Sleep Quality Index[Title/abstract]) OR (Epworth Sleepiness Scale[Title/abstract]) OR (Subjective sleep quality[Title/abstract]) OR (Sleep latency[Title/abstract]) OR (Sleep duration[Title/abstract]) OR (Habitual sleep efficiency[Title/abstract]) OR (Sleep disturbances[Title/abstract]) OR (Use of sleeping medication[Title/abstract]) OR (Daytime dysfunction[Title/abstract]) OR (severity of sleep onset[Title/abstract]) OR (sleep maintenance[Title/abstract]) OR (early morning awakening problems[Title/abstract]) OR (sleep dissatisfaction[Title/abstract]) OR (interference of sleep difficulties with daytime functioning[Title/abstract]) OR (noticeability of sleep problems by others[Title/abstract]) OR (distress caused by the sleep difficulties[Title/abstract])) |
| Embase | (‘medical student’/exp OR ‘medical student’:ti,ab OR ‘medical undergraduates’:ti,ab OR ‘medical graduate’/exp OR ‘medical graduate’:ti,ab OR ‘student’:ti,ab)  AND  (‘smartphone usage’:ti,ab OR ‘screentime’:ti,ab OR ‘smartphone addiction scale’/exp OR ‘smartphone addiction scale’:ti,ab OR ‘smartphone addiction scale short version’/exp OR ‘smartphone addiction scale short version’:ti,ab OR ‘smartphone addiction scale short version’/exp OR ‘smartphone addiction scale short version’:ti,ab OR ‘Problematic Use of Mobile Phones’:ti,ab OR ‘Mobile Phone Dependence’:ti,ab OR ‘App Usage Tracker’:ti,ab OR ‘Android Application Quality Time’:ti,ab OR ‘Daily-life disturbance’:ti,ab OR ‘Withdrawal’:ti,ab OR ‘Cyberspace-oriented relationship’:ti,ab OR ‘Overuse’:ti,ab OR ‘Tolerance’:ti,ab OR ‘Prohibited use’:ti,ab OR ‘Dangerous use’:ti,ab OR ‘Dependent use’:ti,ab OR ‘Financial problems resulting from use’:ti,ab OR ‘Smartphone usage duration’:ti,ab OR ‘Tolerance and interference with other activities’:ti,ab OR ‘Abstinence syndrome’:ti,ab OR ‘Impaired impulse control’:ti,ab OR ‘problems derived from use’:ti,ab)  AND  (‘sleep quality’/exp OR ‘sleep quality’:ti,ab OR ‘sleep time’/exp OR ‘sleep time’:ti,ab OR ‘sleep quality scale’/exp OR ‘sleep quality scale’:ti,ab OR ‘Pittsburgh Sleep Quality Index’/exp OR ‘Pittsburgh Sleep Quality Index’:ti,ab OR ‘Epworth sleepiness scale’/exp OR ‘Epworth sleepiness scale’:ti,ab OR ‘Subjective sleep quality’:ti,ab OR ‘Sleep latency’:ti,ab OR ‘Sleep duration’:ti,ab OR ‘Habitual sleep efficiency’:ti,ab OR ‘Sleep disturbances’:ti,ab OR ‘Use of sleeping medication’:ti,ab OR ‘Daytime dysfunction’:ti,ab OR ‘severity of sleep onset’:ti,ab OR ‘sleep maintenance’:ti,ab OR ‘early morning awakening problems’:ti,ab OR ‘sleep dissatisfaction’:ti,ab OR ‘interference of sleep difficulties with daytime functioning’:ti,ab OR ‘noticeability of sleep problems by others’:ti,ab OR ‘distress caused by the sleep difficulties’:ti,ab) |
| **Cinahl** | ((medical students) OR (medical undergraduates) OR (medical undergraduates) OR (medical graduates) OR (student))  AND  ((smartphone usage) OR (screentime) OR (Smartphone Addiction Scale) OR (Problematic Use of Mobile Phones) OR (Mobile Phone Dependence) OR (App Usage Tracker) OR (Android Application Quality Time) OR (Daily-life disturbance) OR (Withdrawal) OR (Cyberspace-oriented relationship) OR (Overuse) OR (Tolerance) OR (Prohibited use) OR (Dangerous use) OR (Dependent use) OR (Financial problems resulting from use) OR (Smartphone usage duration) OR (Tolerance and interference with other activities) OR (Abstinence syndrome) OR (Impaired impulse control) OR (Problems derived from use))  AND  ((sleep quality) OR (sleep duration) OR (Pittsburgh Sleep Quality Index) OR (Epworth Sleepiness Scale) OR (Subjective sleep quality) OR (Sleep latency) OR (Habitual sleep efficiency) OR (Sleep disturbances) OR (Use of sleeping medication) OR (Daytime dysfunction) OR (severity of sleep onset) OR (sleep maintenance) OR (early morning awakening problems) OR (sleep dissatisfaction) OR (interference of sleep difficulties with daytime functioning) OR (noticeability of sleep problems by others) OR (distress caused by the sleep difficulties)) |
| **PsycInfo** | Psychological papers - esp for sleep rsrch  ((medical students[Mesh terms]) OR (medical students[Title/Abstract]) OR (medical undergraduates[Mesh terms]) OR (medical undergraduates[Title/Abstract]) OR (medical graduates[Title/Abstract]) OR (student[Title/Abstract]))  AND  ((smartphone usage[Title/abstract]) OR (screentime[Title/abstract]) OR (Smartphone Addiction Scale[Title/abstract]) OR (Problematic Use of Mobile Phones[Title/abstract]) OR (Mobile Phone Dependence[Title/abstract]) OR (App Usage Tracker[Title/abstract]) OR (Android Application Quality Time[Title/abstract]) OR (Daily-life disturbance[Title/abstract]) OR (Withdrawal[Title/abstract]) OR (Cyberspace-oriented relationship[Title/abstract]) OR (Overuse[Title/abstract]) OR (Tolerance[Title/abstract]) OR (Prohibited use[Title/abstract]) OR (Dangerous use[Title/abstract]) OR (Dependent use[Title/abstract]) OR (Financial problems resulting from use[Title/abstract]) OR (Smartphone usage duration[Title/abstract]) OR (Tolerance and interference with other activities[Title/abstract]) OR (Abstinence syndrome[Title/abstract]) OR (Impaired impulse control[Title/abstract]) OR (Problems derived from use[Title/abstract]))  AND  ((sleep quality[Mesh terms]) OR (sleep quality[Title/abstract]) OR (sleep duration[Title/abstract]) OR (Pittsburgh Sleep Quality Index[Title/abstract]) OR (Epworth Sleepiness Scale[Title/abstract]) OR (Subjective sleep quality[Title/abstract]) OR (Sleep latency[Title/abstract]) OR (Sleep duration[Title/abstract]) OR (Habitual sleep efficiency[Title/abstract]) OR (Sleep disturbances[Title/abstract]) OR (Use of sleeping medication[Title/abstract]) OR (Daytime dysfunction[Title/abstract]) OR (severity of sleep onset[Title/abstract]) OR (sleep maintenance[Title/abstract]) OR (early morning awakening problems[Title/abstract]) OR (sleep dissatisfaction[Title/abstract]) OR (interference of sleep difficulties with daytime functioning[Title/abstract]) OR (noticeability of sleep problems by others[Title/abstract]) OR (distress caused by the sleep difficulties[Title/abstract])) |

| Cochrane | | |  |
| --- | --- | --- | --- |
|  | Population | Intervention | Outcome |
|  | #1: MeSH Descriptor: [Students, medical] explode all trees  #2: MeSH Descriptor: [Education, Medical, Undergraduate] explode all trees  #3: (Students, Medical):ti,ab,kw  #4: (Education, Medical, Undergraduate):ti,ab,kw | #5: MeSH Descriptor: [Cell Phone Use] explode all trees  #6: MeSH Descriptor: [Internet Addiction Disorder] explode all trees  #7: (Cell Phone Use):ti,ab,kw  #8: (Internet Addiction Disorder):ti,ab,kw  #9: (Smartphone Usage):ti,ab,kw  #10: (Screentime):ti,ab,kw  #11: (Smartphone Addiction Scale):ti,ab,kw  #12: (Problematic Use of Mobile Phones):ti,ab,kw  #13: (Mobile Phone Dependence):ti,ab,kw  #14: (App Usage Tracker):ti,ab,kw  #15: (Android Application Quality Time):ti,ab,kw | #16: (Sleep Quality):ti,ab,kw  #17: (Sleep Time):ti,ab,kw  #18: (Sleep Quality Scale):ti,ab,kw  #19: (Pittsburgh Sleep Quality Index):ti,ab,kw  #20: (Epworth Sleepiness Scale):ti,ab,kw |
| Combined | #21: #1 OR #2 OR #3 OR #4  #22: #5 OR #6 OR #7 OR #8 OR #9 OR #10 OR #11 OR #12 OR #13 OR #14 OR #15  #23: #16 OR #17 OR #18 OR #19 OR #20  #24: #21 AND #22 AND #23 | | |
